# Altered Prefrontal Blood Flow Related With Mild Cognitive Impairment in Parkinson's Disease: A Longitudinal Study

**DOI:** 10.3389/fnagi.2022.896191

**Published:** 2022-07-11

**Authors:** Jian Wang, Wei Zhang, Ying Zhou, Jia Jia, Yuanfang Li, Kai Liu, Zheng Ye, Lirong Jin

**Affiliations:** ^1^Department of Radiology, Zhongshan Hospital, Fudan University, Shanghai, China; ^2^Institute of Neuroscience, Center for Excellence in Brain Science and Intelligence Technology, Chinese Academy of Sciences, Shanghai, China; ^3^Department of Neurology, XiaMen Branch, Zhongshan Hospital, Fudan University, Xiamen, China; ^4^Department of Neurology, Zhongshan Hospital, Fudan University, Shanghai, China

**Keywords:** Parkinson's disease, mild cognitive impairment, arterial spin labeling, longitudinal, prefrontal cortex

## Abstract

Cognitive impairment is a common non-motor symptom in Parkinson's disease (PD), with executive dysfunction being an initial manifestation. We aimed to investigate whether and how longitudinal changes in the prefrontal perfusion correlate with mild cognitive impairment (MCI) in patients with PD. We recruited 49 patients with PD with normal cognition and 37 matched healthy control subjects (HCs). Patients with PD completed arterial spin labeling MRI (ASL–MRI) scans and a comprehensive battery of neuropsychological assessments at baseline (V0) and 2-year follow-up (V1). HCs completed similar ASL–MRI scans and neuropsychological assessments at baseline. At V1, 10 patients with PD progressed to MCI (converters) and 39 patients remained cognitively normal (non-converters). We examined differences in the cerebral blood flow (CBF) derived from ASL–MRI and neuropsychological measures (a) between patients with PD and HCs at V0 (effect of the disease), (b) between V1 and V0 in patients with PD (effect of the disease progression), and (c) between converters and non-converters (effect of the MCI progression) using *t*-tests or ANOVAs with false discovery rate correction. We further analyzed the relationship between longitudinal CBF and neuropsychological changes using multivariate regression models with false discovery rate correction, focusing on executive functions. At V0, no group difference was found in prefrontal CBF between patients with PD and HCs, although patients with PD showed worse performances on executive function. At V1, patients with PD showed significantly reduced CBF in multiple prefrontal regions, including the bilateral lateral orbitofrontal, medial orbitofrontal, middle frontal, inferior frontal, superior frontal, caudal anterior cingulate, and rostral anterior cingulate. More importantly, converters showed a more significant CBF reduction in the left lateral orbitofrontal cortex than non-converters. From V0 to V1, the prolonged completion time of Trail Making Test-B (TMT-B) negatively correlated with longitudinal CBF reduction in the right caudal anterior cingulate cortex. The decreased accuracy of the Stroop Color-Word Test positively correlated with longitudinal CBF reduction in the left medial orbitofrontal cortex. In addition, at V1, the completion time of TMT-B negatively correlated with CBF in the left caudal anterior cingulate cortex. Our findings suggest that longitudinal CBF reduction in the prefrontal cortex might impact cognitive functions (especially executive functions) at the early stages of PD.

## Introduction

Cognitive impairment occurs at the early stages of Parkinson's disease (PD) and impacts the quality of life (Aarsland et al., 2017). The spectrum of cognitive impairment in PD encompasses subjective cognitive decline, mild cognitive impairment (PD–MCI), and dementia (PDD) (Aarsland et al., 2021). Approximately one-third of the newly diagnosed patients with PD exhibit PD–MCI (Broeders et al., 2013). About 38% of patients with PD with normal cognition convert to PD–MCI or PDD over 3 years (Nicoletti et al., 2019). Executive dysfunction is an initial manifestation of PD–MCI, including deficits in cognitive flexibility and inhibitory control (Svenningsson et al., 2012). The prefrontal cortex encompasses the lateral prefrontal cortex, medial prefrontal cortex, and orbitofrontal cortex, which play a key role in executive cognitive functions (Jones and Graff-Radford, 2021). Dopaminergically mediated fronto-striatal executive impairment is to be considered partly accountable for cognitive deficit in PD (Kehagia et al., 2013). In this study, we aimed to investigate whether and how longitudinal changes in the prefrontal perfusion correlate with the progression of PD–MCI and executive dysfunction.

Cognitive impairment in PD is associated with cortical hypoperfusion, in addition to other cortical structure and function changes (Melzer et al., 2012; Al-Bachari et al., 2014; Wang et al., 2018). Cortical perfusion is often measured as cerebral blood flow (CBF) derived from the arterial spin labeling (ASL) MRI (Williams et al., 1992). CBF is thought to reflect neural activity and brain glucose metabolism at the capillary level (Musiek et al., 2012). Widespread CBF reduction in the prefrontal cortex, hippocampus, parietal cortex, and precuneus has been linked to global cognitive decline in PD (Fernandez-Seara et al., 2012; Al-Bachari et al., 2014). The frontal lobe has been reported to be associated with executive functions (Fuster, 2000). Patients with PDD showed reduced left inferior frontal CBF, compared with patients with PD with MCI or normal cognition (Azamat et al., 2021). CBF reductions in the right superior frontal gyrus, right middle frontal gyrus, and right anterior and medial orbitofrontal cortex are associated with global cognitive impairment in patients with PD (Liu et al., 2021). Moreover, CBF reduction in the left middle frontal gyrus is associated with executive dysfunction (Suo et al., 2019). Although many cross-sectional studies have demonstrated that lower CBF in patients with PD (relative to healthy adults) is associated with cognitive impairment, longitudinal studies allow us to look at cognitive decline over time in patients with PD. Confounding factors such as age, education, and socioeconomic status can be better controlled in longitudinal studies. In addition, frontal CBF predicts cognitive decline in a healthy aging cohort with 4-year follow-up (De Vis et al., 2018). Hence, the changes in frontal CBF associated with cognitive status alternations in patients with PD is worth verifying.

Therefore, in the longitudinal study herein, we aimed to investigate whether and how prefrontal CBF changes correlate with mild cognitive decline in patients with PD over 2 years. Patients with PD with normal cognition completed ASL–MRI scans and a comprehensive battery of neuropsychological assessments (executive functions, attention/working memory, visuospatial functions, memory, and language) at baseline (V0) and 2-year follow-up (V1). Matched healthy control subjects (HCs) were assessed once at baseline. First, we wanted to detect group differences in prefrontal CBF and neuropsychological measures between patients with PD and HCs at V0 (effect of the disease). Second, we wanted to examine whether prefrontal CBF decreased between V1 and V0 (effect of the disease progression). Third, we separated patients with PD who were converted to MCI (converters) and those who stayed cognitively normal at V1 (non-converters). We aimed to examine whether prefrontal CBF decreased more in converters than non-converters (effect of the MCI progression). Finally, we sought to identify relationships between prefrontal CBF reduction and executive dysfunction. In particular, we asked whether prefrontal CBF changes correlated with changes in the neuropsychological measures of cognitive flexibility or inhibitory control.

## Methods and Materials

This study was approved by the ethics committee of the Fudan University Zhongshan Hospital, according to the Declaration of Helsinki. Before participating in this study, each participant signed a written informed consent.

### Study Design

It is a longitudinal study. In total, 49 patients with PD with normal cognition were recruited and measured at baseline (V0). All the patients completed ASL–MRI scans and a comprehensive battery of neuropsychological assessments. After 2 years (V1), 10 patients with PD converted to MCI (converters), and 39 patients stayed cognitively normal (non-converters). In total, 37 HCs completed similar ASL–MRI scans and neuropsychological assessments at V0.

### Patients and Clinical Assessments

In total 49 patients with PD were recruited from the movement disorders clinically at the Zhongshan Hospital from September 2016 to October 2018. Inclusion criteria included the following: (1) PD diagnosis, according to the Movement Disorder Society clinical diagnostic criteria for PD (Postuma et al., 2015); (2) cognitively normal at V0, not meeting the diagnostic criteria of PDD (Emre et al., 2007) or level 2 criteria of PD–MCI (i.e., 1.5 SDs below the normative score) (Litvan et al., 2012); (3) Hoehn and Yahr stages 1–2; (4) age 40–80 years; and (5) education >6 years. Exclusion criteria included the following: (1) a history of other neurologic or psychiatric diseases; (2) alcohol or drug abuse; or (3) contraindication to MRI. Six additional patients were excluded from data analysis because of excessive head motion during scanning (*n* = 1), MRI artifacts (*n* = 3), or poor MRI co-registration (*n* = 2).

### Healthy Control Subjects

In total, 37 HCs were recruited from the local community. Inclusion criteria included the following: (1) age 40–80 years and (2) education >6 years. Exclusion criteria included the following: (1) a history of significant neurologic or psychiatric disorders; (2) a family history of PD, essential tremor, or other movement disorders; or (3) contraindication to MRI. In total, three additional HCs were excluded from data analysis due to MRI artifacts.

### Neuropsychological Assessments

Neuropsychological tests measured executive functions (Stroop Color and Word Test, Trail Making Test-B), attention/working memory (Symbol Digit Modalities Test, Trail Making Test-A), visuospatial functions (Rey-Osterrieth Complex Figure Test, Clock-Drawing Test), memory (Auditory Verbal Learning Test, delayed recall of the Rey-Osterrieth Complex Figure Test), and language (Animal Fluency Test, Boston Naming Test).

First, we detected group differences between patients with PD and HCs at V0 using two-sample *t*-tests or the Mann–Whitney *U* tests, if the data were not normally distributed (*p* < 0.05 false-discovery-rate-corrected, FDR-corrected). Second, we detected differences between V0 and V1 in patients with PD using paired *t*-tests or the Wilcoxon signed-rank tests, if the data were not normally distributed (*p* < 0.05 FDR-corrected). Third, we detected subgroup differences between converters and non-converters using two-sample *t*-tests or the Mann–Whitney *U* tests, if the data were not normally distributed (*p* < 0.05, FDR-corrected).

### Acquisition of T1-MRI and ASL–MRI Data

Brain imaging data were acquired on a GE Discovery MR750 3T MR scanner. High-resolution T1-weighted images used a 3D brain volume imaging (BRAVO) sequence (136 axial slices; time of repetition, 8.2 ms; time of echo, 3.2 ms; inversion time, 450 ms; field-of-view, 240 × 240 mm; slice thickness, 1.0 mm; no intersection gap; matrix, 256 × 256; number of excitations, 1; flip angle, 12°; bandwidth, 31.25 kHz).

Arterial spin labeling images used a three-dimensional pseudo-continuous ASL (3D pCASL) technique (Ding et al., 2014) with background suppression and outward-direction spiral readout (time of repetition, 4,830 ms; time of echo, 10.5 ms; labeling duration, 1,500 ms; post labeling delay, 2,025 ms; field-of-view, 240 × 240 mm; slice thickness, 4 mm; matrix, 128 × 128; number of excitations, 3; flip angle, 155°; eight spiral arms with 512 points in each arm; bandwidth, 62.5 kHz; the resolution, 1.9 × 1.9 mm). An additional proton density-weighted image of absolute CBF quantification used the same acquisition parameters. Then, ASL images were transferred to the Advantage Workstation for Diagnostic Imaging 4.6 (GE Medical Systems, Milwaukee, WI, USA), and the quantitative CBF maps, in units of ml/100 g/min, were calculated using vender provided toolbox. There were no user-modifiable parameters for generating CBF maps in the toolbox for ASL images.

### Preprocessing and Analysis of MRI Data

MRI data were preprocessed and analyzed using Freesurfer v7.1.0 (Dale et al., 1999; Fischl et al., 2002; Reuter et al., 2012) and FMRIB Software Library v6.0 (Parker Jones et al., 2018) with a standard pipeline. T1 images were corrected for head motion, normalized to reduce signal intensity, non-uniformity, and fluctuation, and transformed into the Talairach space. Non-brain tissues (e.g., skull and neck) were removed. White matter and deep grey matter nuclei were segmented using an automated algorithm. Regions of interest were parcellated according to the Desikan–Killiany atlas, and their tissue volumes were computed. We monitored the potential effects of tissue volume. First, we examined whether patients with PD showed smaller prefrontal volumes than HCs at V0 and whether converters showed smaller prefrontal volumes than non-converters at V1 using two-sample *t*-tests (FDR-corrected). Second, we examined whether patients with PD showed smaller prefrontal volumes at V1 than V0 using paired *t*-tests (FDR-corrected). Third, we examined whether longitudinal changes in the prefrontal volume correlated with those in the prefrontal CBF (FDR-corrected).

### Analysis of Cortical CBF and Statistical Analyses

**Figure 1:**
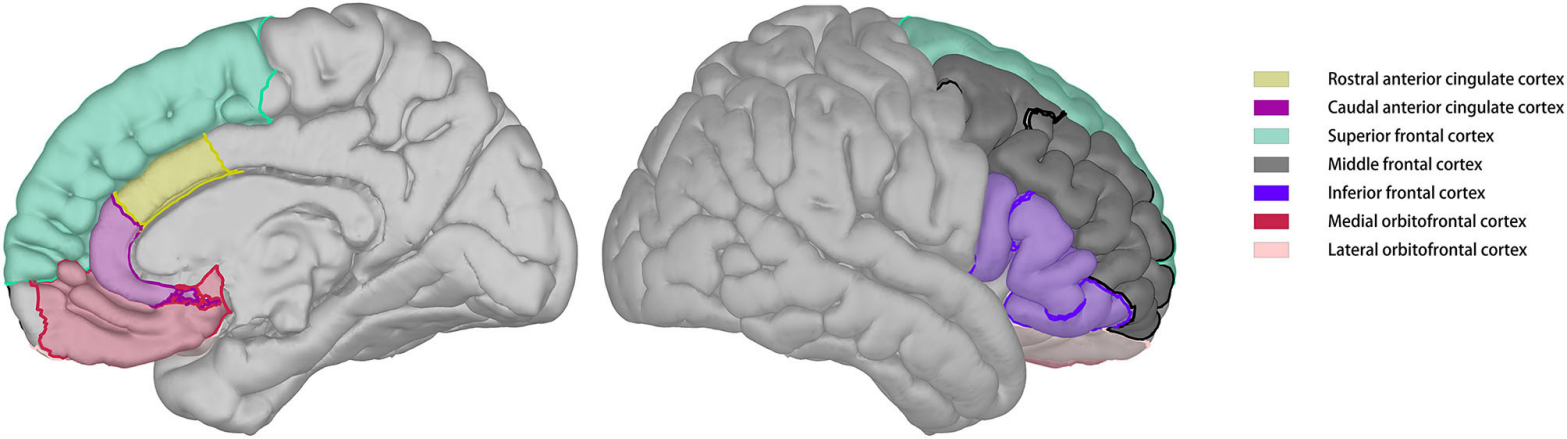
ROIs distribution on brain model.

Cerebral blood flow images were skull-stripped and automatically co-registered with reconstructed T1 images. Co-registration results were visually inspected and manually adjusted to ensure quality. Regional CBF was calculated according to the parcellation of T1 images. The regions of interest for this study included the lateral orbitofrontal cortex, medial orbitofrontal cortex, superior frontal cortex, middle frontal cortex, inferior frontal cortex, caudal anterior cingulate cortex, and rostral anterior cingulate cortex of both the hemispheres (Figure 1).

First, we examined whether patients with PD showed lower prefrontal CBF than HCs at V0 using two-sample *t*-tests or the Mann–Whitney *U* tests, if the data were not normally distributed (*p* < 0.05, FDR-corrected). Second, we examined whether patients with PD showed lower prefrontal CBF at V1 than V0 using paired *t*-tests or the Wilcoxon signed-rank tests if the data were not normally distributed (*p* < 0.05, FDR-corrected). Third, we explored whether converters showed a more significant prefrontal CBF reduction than non-converters using repeated-measures ANOVAs (*p* < 0.05) with two factors, subgroups (converters and non-converters) and visit (V0 and V1). Forth, we identified the relationship between prefrontal CBF and neuropsychological measures of executive functions using stepwise linear regressions (*p* < 0.05, FDR-corrected). In particular, we asked whether longitudinal changes in the completion time of TMT-B or accuracy of Stroop Test between V1 and V0 correlated with prefrontal CBF changes, prefrontal volume changes, UPDRS motor score changes, depression score changes, cortical volume changes, age changes, or education. In addition, we asked whether the completion time of TMT-B or accuracy of Stroop Test at V0 or V1 correlated with prefrontal CBF, when prefrontal volume, UPDRS motor scores, depression scores, age, and education were controlled.

## Results

### Group Differences and Longitudinal Changes in Neuropsychological Measures

Table 1 shows neuropsychological data of patients with PD and HCs. At V0, patients with PD performed worse than HCs on executive functions and working memory. However, no significant longitudinal changes were found between V0 and V1. Table 2 illustrates neuropsychological data of converters and non-converters. Converters performed worse than non-converters on executive functions, visuospatial functions, and language at V1 but not V0. Converters performed worse than non-converters on working memory and memory at both visits.

**Table 1:** Demographic, clinical, and neuropsychological data of PD patients and HCs (means, SDs, and statistic differences).

**Features/Measures**	**HC (*n* = 37)**	**PD (*****n*** **= 49)**		**Statistic differences (*****p*** **values)**	
		**V0**	**V1**	**PD at V0 vs. HC**	**PD at V1 vs. V0**
Male/Female	15/22	26/23	26/23	0.157	–
Age (years)	62.2 (6.9)	63.8 (9.0)	65.8 (9.0)	0.373	–
Education (years)	12.2 (2.6)	12.0 (3.3)	12.0 (3.3)	0.430	–
Disease duration (months)		42.7 (69.0)	66.9 (68.4)	–	–
Motor symptoms					
Hoehn and Yahr stage		1.3 (0.5)	1.6 (0.6)	–	0.005^*^
UPDRS III		16.7 (7.5)	18.7 (8.4)	–	0.011^*^
Non-motor symptoms					
REM Sleep Behavior Disorder Screening Questionnaire score		3.6 (3.4)	4.6 (3.0)	–	0.028
Geriatric Depression Scale score (30-items)		7.8 (6.4)	10.9 (5.4)	–	0.002^*^
Executive functions					
Stroop (completion time)	66.7 (17.2)	76.7 (19.0)	77.6 (22.0)	0.013^*^	0.604
Stroop (number of correct answers)	48.0 (2.3)	47.2 (3.1)	46.5 (3.4)	0.297	0.114
TMT-B (completion time)	111.3 (39.9)	138.8 (37.9)	144.1 (41.9)	0.002^*^	0.309
Attention/working memory					
Symbol Digit Modalities Test score	47.7 (10.3)	40.5 (10.3)	38.3 (10.2)	0.002^*^	0.040
TMT-A (completion time)	52.2 (20.2)	60.4 (17.1)	58.9 (19.5)	0.043	0.379
Visuospatial functions					
Rey-Osterrieth Complex Figure Test score	32.9 (2.3)	32.4 (2.5)	31.9 (3.0)	0.441	0.126
Clock Drawing Test score	9.3 (0.7)	9.4 (0.8)	9.1 (1.5)	0.644	0.292
Memory					
Auditory Verbal Learning Test total score	32.4 (7.9)	31.2 (9.5)	32.5 (12.3)	0.540	0.283
Rey-Osterrieth Complex Figure Test delayed recall score	16.8 (6.5)	16.7 (7.0)	15.6 (6.9)	0.901	0.094
Language					
Animal Fluency Test score	18.6 (4.9)	19.9 (5.3)	18.8 (5.7)	0.267	0.150
Boston Naming Test score (30-items)	25.7 (2.9)	25.8 (2.4)	25.6 (2.8)	0.965	0.564

*Statistic differences, p values of two-sample t-tests, paired-sample t-tests, Mann-Whitney U tests or Wilcoxon signed-rank tests as appropriate; asterisks, p < 0.05 false-discovery-rate-corrected*.

*UPDRS, Unified Parkinson's Disease Rating Scale; TMT, Trail Making Test*.

**Table 2:** Demographic, clinical, and neuropsychological data of converters and non-converters (means, SDs, and statistic differences).

**Features/Measures**	**V0 (*****n*** **= 49)**		**V1 (*****n*** **= 49)**		**Subgroup differences (*****p*** **values)**	
	**Non-converters**	**Converters**	**Non-converters**	**Converters**	**V0**	**V1**
Male/Female	21/18	5/5	21/18	5/5	0.157	0.157
Age (years)	63.1 (9.6)	66.4 (5.8)	65.1 (9.6)	68.5 (5.8)	0.301	0.301
Education (years)	12.6 (3.2)	9.3 (1.9)	12.6 (3.2)	9.3 (1.9)	0.005^*^	0.005^*^
Disease duration (months)	45.3 (75.2)	32.5 (36.4)	69.8 (75.4)	58.0 (35.5)	0.634	0.634
Motor symptoms						
Hoehn and Yahr stage	1.3 (0.4)	1.6 (0.5)	1.5 (0.6)	1.8 (0.6)	0.039	0.163
UPDRS III	15.4 (6.1)	21.5 (10.5)	17.9 (7.6)	21.8 (11.0)	0.117	0.194
Non-motor symptoms						
REM Sleep Behavior Disorder Screening Questionnaire score	3.6 (3.5)	3.8 (3.0)	4.5 (3.1)	4.8 (3.1)	0.862	0.792
Geriatric Depression Scale score (30-items)	7.2 (5.9)	10.1 (7.9)	10.3 (5.1)	13.1 (6.4)	0.197	0.144
Executive functions						
Stroop (completion time)	74.3 (19.1)	86.2 (15.8)	74.0 (20.5)	98.9 (21.5)	0.076	0.001^*^
Stroop (number of correct answers)	47.2 (3.3)	47.3 (2.4)	47.3 (2.6)	42.6 (4.2)	0.914	<0.001^*^
TMT-B (completion time)	133.7 (37.7)	158.9 (35.1)	133.2 (35.6)	186.0 (37.9)	0.060	<0.001^*^
Attention/working memory						
Symbol Digit Modalities Test score	42.6 (10.0)	32.1 (6.2)	40.5 (9.8)	29.9 (6.9)	0.003^*^	0.003^*^
TMT-A (completion time)	59.4 (17.8)	64.3 (14.1)	54.1 (15.9)	77.7 (21.5)	0.427	<0.001^*^
Visuospatial functions						
Complex Figure Test score	32.8 (2.1)	30.7 (3.2)	17.4 (6.2)	28.8 (3.7)	0.018	0.002^*^
Clock Drawing Test score	9.5 (0.6)	8.9 (1.2)	9.2 (1.4)	8.7 (1.8)	0.041	0.308
Memory						
Auditory Verbal Learning Test total score	32.4 (10.0)	26.2 (4.9)	35.1 (11.5)	22.1 (9.9)	0.039	0.002^*^
Rey-Osterrieth Complex Figure Test delayed recall score	18.5 (6.1)	9.6 (5.2)	17.4 (6.2)	8.2 (3.8)	<0.001^*^	<0.001^*^
Language						
Animal Fluency Test score	16.7 (3.5)	20.8 (5.4)	20.3 (5.1)	13.1 (4.2)	0.030	<0.001^*^
Boston Naming Test score (30-items)	26.0 (2.4)	24.7 (2.2)	26.3 (2.4)	23.0 (2.8)	0.114	<0.001^*^

*Subgroup differences, p values of two-sample t-tests, paired-sample t-tests, Mann-Whitney U tests or Wilcoxon signed-rank tests as appropriate; asterisks, p < 0.05 false-discovery-rate-corrected*.

*UPDRS, Unified Parkinson's Disease Rating Scale; TMT, Trail Making Test*.

### Prefrontal CBF Difference Between Patients With PD at V0 and V1

**Figure 2:**
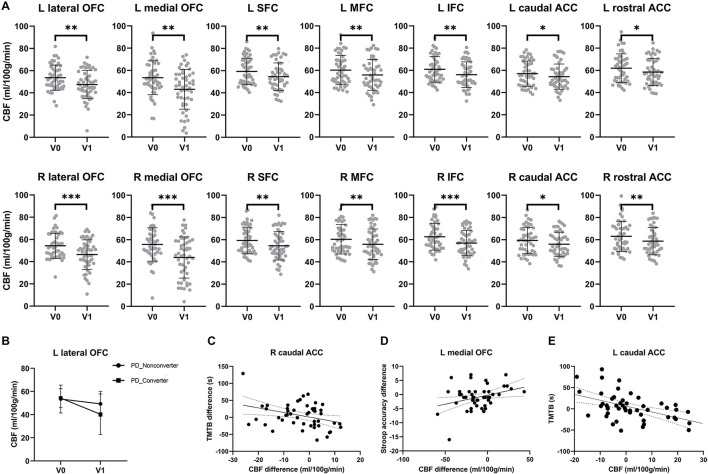
Reduced prefrontal cerebral blood flow and executive dysfunction over time in PD patients. **(A)** Significant CBF reduction in the lateral orbitofrontal cortex, medial orbitofrontal cortex, middle frontal cortex, inferior frontal cortex, superior frontal cortex, caudal anterior cingulate cortex and rostral anterior cingulate cortex of both hemispheres over 2 years. Asterisks indicate significant group difference (*: *p* < 0.05, **: *p* < 0.01, ***: *p* < 0.001). **(B)** Converters showed a more significant perfusion reduction in the left lateral orbitofrontal cortex than non-converters over 2 years. **(C)** The prolonged completion time of Trail Making Test-B (TMT-B) was negatively correlated with longitudinal CBF reduction in the right caudal anterior cingulate cortex. **(D)** Longitudinal CBF reduction in right caudal anterior cingulate cortex was significantly related to the progressive worse accuracy of Stroop test over time. **(E)** At V1, completion time of TMT-B had a significantly negative correlation with the CBF in left caudal anterior cingulate cortex.

No significant difference was found between PD and HC at V0 (*p* > 0.36 for all the ROIs). Figure 2A, Table 3 show decreased prefrontal CBF in patients with PD at V1. Patients with PD showed significantly lower CBF at V1 than V0 in multiple ROIs (*p* < 0.05).

**Table 3:** The prefrontal perfusion significantly different between V0 and V1 in PD patients.

**Region**	***t*** **-value**	***p*** **-value**
Left lateral orbitofrontal cortex	3.52	*p* = 0.002
Right lateral orbitofrontal cortex	4.52	*p < 0.001*
Left medial orbitofrontal cortex	3.57	*p* = *0.001*
Right medial orbitofrontal cortex	3.88	*p < 0.001*
Left middle frontal cortex	2.85	*p* = *0.006*
Right middle frontal cortex	3.01	*p* = *0.004*
Left inferior frontal cortex	3.72	*p* = *0.001*
Right inferior frontal cortex	4.55	*p < 0.001*
Left superior frontal cortex	3.16	*p* = *0.003*
Right superior frontal cortex	3.11	*p* = *0.003*
Left caudal anterior cingulate cortex	2.22	*p* = *0.031*
Right caudal anterior cingulate cortex	2.53	*p* = *0.015*
Left rostral anterior cingulate cortex	2.53	*p* = *0.015*
Right rostral anterior cingulate cortex	3.32	*p* = *0.002*

### Prefrontal CBF Differences Between Converters and Non-converters

In patients with PD, converters showed a more significant CBF reduction (25.8%) than non-converters at V1(7.8%) in the left lateral orbitofrontal cortex (Figure 2B). A marginal subgroup difference was found in the left lateral orbitofrontal cortex, an interaction between subgroup and visit was found [*F*(1,96) = 4.66, *p* = 0.036, partial η^2^ = 0.90] in addition to an effect of visit [*F*(1) = 16.0, *p* < 0.001, partial η^2^ = 0.254]. No main effect of the subgroup was found (*p* = 0.256). No effect of the subgroup was found in any other prefrontal ROIs (*ps* > 0.145).

### Relationships Between Longitudinal CBF Reduction and Cognitive Decline

Figures 2C,D shows relationships between longitudinal CBF reduction and executive dysfunction. The stepwise regression model for cognitive flexibility (i.e., TMT-B completion time difference between V1 and V0) was significant [*F*(1,47) = 6.27, *R*^2^ = 0.118, *p* = 0.016]. The model included longitudinal CBF changes in the right caudal anterior cingulate cortex (*t* = −2.50, *p* = 0.016) but no other factors (*ps* > 0.875). The stepwise regression model for inhibitory control (i.e., Stroop accuracy difference between V1 and V0) was also significant [*F*(1,47) = 5.52, *R*^2^ = 0.105, *p* = 0.023]. The model included longitudinal CBF changes in the left medial orbitofrontal cortex (*t* = 2.35, *p* = 0.023) but no other factors (*ps* > 0.737). No such correlation was obtained for the TMT-A completion time difference (*p* = 0.569).

Figure 2E shows relationships between prefrontal CBF and cognitive flexibility at V1. The stepwise regression model for the TMT-B completion time was significant [*F*(4,44) = 17.3, *R*^2^ = 0.536, *p* < 0.001]. The model included CBF in the left caudal anterior cingulate cortex (*t* = −3.71, *p* = 0.001), education (*t* = −3.07, *p* = 0.004), and age (*t* = 4.23, *p* < 0.001) but no other factors (*ps* > 0.052). No such correlation was obtained for the TMT-A completion time. No significant correlation was found for either the TMT-A or TMT-B completion time at V0.

### Effects of Tissue Volume

Finally, we excluded potential effects of tissue volume. Patients with PD were similar as HCs at V0 in the total intracranial volume (*p* = 0.856), total cortical grey matter volume (*p* = 0.885), the volume of prefrontal ROIs (*ps* > 0.173). Converters were similar to non-converters at V1 in the total intracranial volume (*p* = 0.452), total cortical grey matter volume (*p* = 0.737), and the volume of prefrontal ROIs (*ps* > 0.150). However, from V0 to V1, subjects with PD showed a significantly cortical atrophy in the bilateral lateral orbitofrontal cortex (left: *t*(48) = 2.91, *p* = 0.005; right: *t*(48) = 2.98, *p* = 0.005), left medial orbitofrontal cortex (*t*(48) = 2.65, *p* = 0.011), left superior frontal cortex (*t*(48) = 2.97, *p* = 0.005), bilateral middle frontal cortex (left: *t*(48) = 2.98, *p* = 0.005; right: *t*(48) = 3.19, *p* = 0.002), bilateral inferior frontal cortex (left: *t*(48) = 2.48, *p* = 0.017; right: *t*(48) = 3.62, *p* < 0.001), and bilateral rostral anterior cingulate cortex (left: *t*(48) = 2.63, *p* = 0.011; right: *t*(48) = 2.82, *p* = 0.007). Importantly, longitudinal changes in the prefrontal CBF did not correlate with those in the prefrontal volume (*ps* > 0.369).

## Discussion

Mild cognitive impairment is a common non-motor symptom in PD. Approximately 28% of patients with PD converted to PD–MCI and 6% converted to PDD over 1–16 years (Saredakis et al., 2019). However, biological mechanisms underlying the progression of cognitive impairment are still unclear. This longitudinal study investigated whether and how prefrontal perfusion changes correlate with mild cognitive decline in patients with PD using ASL–MRI. Patients with PD with normal cognition showed normal prefrontal perfusion at baseline but worse performance on executive functions than healthy controls. As the disease progressed, patients with PD showed decreased perfusion in multiple prefrontal regions over 2 years (effect of the disease progression). More importantly, patients with PD who converted to MCI showed a more significant perfusion reduction in the left lateral orbitofrontal cortex than those who stayed cognitively normal at 2-year follow-up (effect of the MCI progression). In particular, the impairment of cognitive flexibility (reflected as the prolonged completion time of TMT-B) correlated with perfusion reduction in the right caudal anterior cingulate cortex, and the impairment of inhibitory control (reflected as the fewer correct responses of the Stroop Test) correlated with perfusion reduction in the left medial orbitofrontal cortex. Although patients with PD showed additional cortical atrophy in the prefrontal cortex, prefrontal volumetric reduction did not significantly contribute to the progression of MCI or executive dysfunction. These findings suggest that longitudinal prefrontal perfusion reduction may be underlying the progression of cognitive impairment at the early stages of PD.

Parkinson's disease is a neurodegenerative and progressive disorder. However, autopsy studies are not sufficient to discover the underlying causes of the progression of PD-related pathology, especially in the early stages. Neuroimaging studies provide an alternative way that might help to reveal the pathophysiology of disease progression. In the present study, at baseline, no group difference was found in prefrontal CBF between patients with PD and HCs, while reduced CBF was shown in the bilateral lateral and medial orbitofrontal cortex, bilateral inferior, middle, and superior cortex and caudal, rostral anterior cingulate cortex over 2 years in patients with PD *via* ASL–MRI. A primary novel finding is that the converters showed progressive hypoperfusion in the left lateral orbitofrontal gyrus, compared with the non-converters between V0 and V1. In addition, there were subtle differences between converters and non-converters at V0 in attention/working memory and memory. Our result was also consistent with Wilsen et al., which showed severer motor symptoms and poor performance on cognitive tests were associated with cognitive decline at the early stages of PD (Wilson et al., 2020). Overall, our results indicated the serial reduction of frontal perfusion along with disease progression and partly contribute to cognitive decline in the early stage of PD. The mechanisms/pathophysiology of cognitive decline in PD is not fully clear, and it is generally considered that multiple degenerations of neurotransmitter systems were associated with cognitive impairment (Aarsland et al., 2021). In addition to the fundamental pathological changes associated with cognitive impairment, some previous studies report cerebral perfusion deficiencies in PD (Fernandez-Seara et al., 2012; Al-Bachari et al., 2014), including our study. It is not known how CBF influences cognitive function. Several reviews summarize that hypoperfusion leads to cognitive impairment partly because of the neurovascular unit dysfunction, disrupted neurotransmitter circuits, and mitochondrial energy deficiency (Iadecola, 2017; de la Torre et al., 2020). One review demonstrated cerebral hypoperfusion, and glucose hypometabolism trigger neuroinflammation and oxidative stress that in turn decrease nitric oxide, upregulating amyloid, and tau in AD (Daulatzai, 2017). Brain perfusion was correlated with cerebrospinal fluid Aβ42 levels which reflected neurodegeneration in patients with amnestic MCI (Quattrini et al., 2021). Moreover, chronic cerebral hypoperfusion *via* stenosis of the bilateral common carotid arteries may exacerbate cognitive impairment in a mouse model of PD (Tang et al., 2017). To some extent, the current study might aid our understanding of the pathophysiology of cognitive conversion in PD.

In addition, neuroimaging features that can track changes in brain structure and functions and associate with neurobiological processes might be applied as longitudinal progression markers in clinical use in PD. Using dopamine transporter imaging, Kaasinen et al. reported progressive striatal dopamine loss (Kaasinen and Vahlberg, 2017). However, the cost, availability, and ceiling effect of dopaminergic deficit limited the use of dopamine transporter imaging in the follow-up study. In total, two recent longitudinal studies demonstrated serial neuromelanin signal loss in substantia nigra (SN), and associated were with changes in motor severity in patients with PD (Gaurav et al., 2021; Xing et al., 2022). Even so, SN neuronal loss might not play a key role in the pathophysiology of non-motor symptoms in PD. To date, several studies using voxel-based morphometry analyses have reported alternation of gray volume and thickness with follow-up period, but inconsistent results indicated that this biomarker was not sensitive to reflect small changes in the disease progression (Yang et al., 2018). Our longitudinal data suggested prefrontal CBF reduction over time and related to cognitive decline in PD, which might be a potential imaging marker associated with cognitive impairment. Along with the neuroimaging biomarker, we note that some neuropsychological tests were reported to be a predictor of developing PDD in the longitudinal cohort (Kim et al., 2019; Lawson et al., 2021). Byeon H also reported a random forest model including MMSE total score, MoCA total score, UPDRS motor score, and Clinical Dementia Rating (CDR) sum of boxes that can predict PD–MCI with an overall accuracy of 65.6% (Byeon, 2020). Since ASL–MRI is relatively low-cost neuroimaging and can be repeated many times for its non-invasion to quantify CBF objectively and quickly, we thought neuropsychological tests, together with an imaging marker or blood/ CSF-based marker might be more sensitive and accurate to predict PD–MCI and PDD in the future study. On the contrary, our results inferred that patients with PD should avoid cerebral hypoperfusion which might in turn cause disease progression in the clinical practice.

Executive dysfunction occurs at a relatively higher frequency in PD–MCI (Zalonis et al., 2008; Baiano et al., 2020). Our longitudinal analyses revealed that over 2 years, reduction of prefrontal CBF in the medial orbitofrontal cortex and caudal anterior cingulate cortex have a significant correlation with poorer performance on inhibitory control and cognitive flexibility, bypassing the potential confounding effects of tissue volume. This could suggest that the prefrontal hypoperfusion might partly be involved in executive dysfunction. High-level cognitive abilities involve cortical regions. TMT-B tests provide a measure of cognitive function associated with the working memory, sequencing, and set-shifting (Sánchez-Cubillo et al., 2009), while Stroop measures executive inhibition of irrelevant responses (Treisman and Fearnley, 1969). Consistently, Brück et al. revealed performance in the Stroop test negatively correlated with the Fdopa uptake in the medial frontal cortex of early PD using positron emission tomography scanning (Brück et al., 2005). Moreover, in an animal study, impaired cognitive flexibility may be partly because of the disruption of the anterior cingulate cortex and its neuromodulation with thalamic nuclei (Bubb et al., 2021). Further studies would be required to detect the role of the prefrontal cortex in large-scale networks or neural circuits.

A previous report by Zhou et al. showed progressive bilateral superior frontal lobe atrophy was found in the converters (Zhou et al., 2020). In the present study, significant prefrontal cortical atrophy was not found compared with the HCs at baseline, and significantly decreased between V1 and V0 in some prefrontal regions. However, we also found significant CBF reduction in the right medial orbitofrontal, right superior frontal, and bilateral caudal anterior cingulate cortex, while no cortical atrophy was observed. Furthermore, there were no correlations between the changes in cortical CBF and volume. Some cross-sectional studies report that the patients with PD showed hypoperfusion in the parietal lobule or occipital lobe with the absence of gray matter volume loss (Pelizzari et al., 2020). Combining our results and the abovementioned studies, it might suggest that functional changes may potentially be involved in neurodegeneration, independent of gray matter atrophy. Notably, there is evidence that insufficient CBF made a chronic and cumulative effect on brain atrophy (de la Torre, 2012). It would be interesting to test in future studies whether CBF reduction contributes to brain atrophy or synchronize with volume loss in the early stage of PD.

Moreover, our data suggested that CBF was not significantly different between HCs and early-stage PD at baseline. This result is consistent with Al-Bachari et al. (2014), but differs from the majority of studies (Derejko et al., 2006; Arslan et al., 2020; Liu et al., 2021), which showed decreased cortical perfusion in the early-stage PD cohort, compared with the healthy controls. This discrepancy may be partly due to the subjects recruited in the studies, as only patients with PD with the normal condition were recruited in our study. In addition, differences in the scanning sequence and measurement methods might be the reasons for inconsistent results.

Several limitations should be recognized in our study. First, the small patient pool subsequently led to fewer patients in the converter subgroup, thus, probably resulting in bias. The intrinsic difference of converter in both CBF and cognitive function may be revealed with a larger sample group, and the data from the PD–MCI cohort may also help to explain the differences. In addition, there seems to be a reasonable chance of types 1 and 2 errors in a small sample size. However, since this is a pilot longitudinal exploration studying the association between changes in CBF and cognition decline, we focused on sample quality rather than the sample size. Future studies, ideally from multiple centers and with more patients recruited, might help to confirm the results of our study and generalize to individuals. Second, a time-match follow-up data acquisition was not performed in HCs, which may provide information on the CBF and cognitive function changes under the effect of aging. Further study should recruit healthy controls, along with the data on patients with PD.

In conclusion, this study demonstrated that longitudinal changes in the prefrontal perfusion underlay the progression of cognitive impairment at the early stages of PD. Patients with PD with normal cognition did not show prefrontal hypoperfusion at baseline, but significant perfusion reductions as the disease progressed over 2 years. More importantly, patients with PD who were converted to MCI showed a more significant perfusion reduction in the left lateral orbitofrontal cortex than those who stayed cognitively normal over 2 years. In particular, declines in cognitive flexibility and inhibitory control were associated with perfusion reductions in the right caudal anterior cortex and the left medial orbitofrontal cortex, respectively.

## Data Availability Statement

The original contributions presented in the study are included in the article/supplementary material, further inquiries can be directed to the corresponding author/s.

## Ethics Statement

The studies involving human participants were reviewed and approved by Ethics Committee of Fudan University Zhongshan Hospital. The patients/participants provided their written informed consent to participate in this study. Written informed consent was obtained from the individual(s) for the publication of any potentially identifiable images or data included in this article.

## Author Contributions

LJ: conceptualized and designed the study. JW, YZ, JJ, YL, KL, and LJ: collected data. WZ and LJ: analyzed the data. WZ, ZY, and LJ: wrote the original draft of the manuscript. JW, YZ, JJ, YL, and KL: edited and reviewed the manuscript. All authors approved the submitted version.

## Funding

This work was supported by the National Natural Science Foundation of China (31961133025). LJ was supported by the State Key Laboratory of Neuroscience (SKLN-202107).

## Conflict of Interest

The authors declare that the research was conducted in the absence of any commercial or financial relationships that could be construed as a potential conflict of interest.

## Publisher's Note

All claims expressed in this article are solely those of the authors and do not necessarily represent those of their affiliated organizations, or those of the publisher, the editors and the reviewers. Any product that may be evaluated in this article, or claim that may be made by its manufacturer, is not guaranteed or endorsed by the publisher.
